# Zebra Finches As a Model Species to Understand the Roots of Rhythm

**DOI:** 10.3389/fnins.2016.00345

**Published:** 2016-07-22

**Authors:** Michelle J. Spierings, Carel ten Cate

**Affiliations:** ^1^Behavioural Biology, Institute Biology Leiden, Leiden UniversityLeiden, Netherlands; ^2^Leiden Institute for Brain and Cognition, Leiden UniversityLeiden, Netherlands

**Keywords:** zebra finches, rhythm per, cognition, song system, vocal learning

## Vocal timing in zebra finches

Zebra finches are a widely used model species for neurobehavioral research, in particular in relation to song development and auditory processing. Males learn their songs from a tutor. Females don't sing, but do develop learned song preferences. Regardless of the differences, both sexes exchange calls in social interactions. Two fascinating recent studies looked at different aspects of rhythmicity in the production of zebra finch vocalizations (Benichov et al., 2016a; Norton and Scharff, 2016), and together with several studies on the perception of rhythms, they make the zebra finch a promising model species to unravel the roots of rhythm production and perception.

Norton and Scharff (2016) analyzed the intervals from one note onset to the next one in both isolate and directed zebra finch songs. For each male they were able to derive an isochronous sequence of “time stamps” of which a subset aligned with all note onsets of a male's song. Moreover, these time stamps often also coincided with the transitions between phonetically different gestures within a complex note. This indicates that an isochronous rhythm might underlie zebra finch songs. Benichov et al. (2016a,b) showed that both males and females can dynamically adjust the mutual timing of their calls. Individual birds were housed with a robotic zebra finch that emitted an isochronous call pattern. Within ample minutes the zebra finches adjusted their call rate to create a regular back-and-forth exchange with the robotic finch. The robot was then set to emit “jamming” calls that were produced at the moment when the zebra finch was most likely to respond. All zebra finches adjusted their call pattern to avoid the jamming calls, either by calling earlier, later or both earlier and later. Furthermore, when the robotic finch produced a pattern of alternating single and paired calls, the zebra finches timed their calls differently for the single compared to the paired calls, indicating they apparently detected the alternating pattern of the robot calls and used this to anticipate whether the next call would be single or paired. Interestingly, females performed better than males at these tasks (Benichov et al., 2016a).

Benichov et al. (2016a) next examined the role of the forebrain song system in the timing of calls. The female song system is reduced compared to the male system and lacks, for instance, the largest song nucleus, Area X. However, some nuclei of the song system are present in both sexes, like the RA, a nucleus related to the temporal aspects of zebra finch song learning. Lesioning the RA nucleus in zebra finches made them unable to correctly adjust the timing of their calls, although they were still responsive to the robotic finch. This indicates that for both males and females the RA is actively involved in call synchronization and vocal coordination. Further experiments showed that input to the RA from another forebrain nucleus, the HVC, was necessary to maintain call synchronization (Benichov et al., 2016a). While Norton and Scharff (2016) did not examine the neural basis of the isochronous patterns they observed in male songs, they also suggest that it might originate from the activation pattern of HVC neurons firing to the RA. These HVC_RA_ neurons fire in a rhythmic, clock-like pattern (Long and Fee, 2008). However, as the rhythms observed by Norton and Scharff are 3 to 10 times slower than the firing of these HVC neurons, they propose that additional mechanisms must operate to translate the clocklike firing into the higher complexity observed in the songs.

Whether animals can perceive beat and rhythmic patterns is a prominent question in relation to understanding the evolution of human musicality (Patel, 2006, 2014; Fitch, 2013; Hoeschele et al., 2015; Honing et al., 2015) and Benichov et al. (2016a,b) suggest that the link between the neural system involved in vocal learning and call synchronization provides the relation between beat perception and auditory-motor coordination as shown in a number of animal species. So, how do the above findings on the presence of rhythms in vocal production in zebra finches relate to studies that addressed the perception of rhythmic auditory patterns?

## Recognizing regularity

Rhythm can be defined as a regular repeated pattern. In its simplest form it is an isochronous series of pulses, like the one produced by the robotic finch. More complicated rhythms can be created by repeating a heterochronous pattern, for example the repetition of single and double pulses as used in Benichov et al. (2016a). Humans are skilled in perceiving various rhythms. We can easily detect whether a pulse or beat pattern is regular or irregular by integrating temporal information over a series of auditory events (e.g., Geiser et al., 2014). We thus have the cognitive ability to abstract a general, global, pattern from a string of sounds, enabling us to classify patterns as regular or not. However, the question whether or to what extent non-human animals are also able to integrate temporal information over a longer series of sounds to classify strings as being regular is still open. A telling example is a study by Hagmann and Cook (2010) that showed that pigeons were unable to learn to discriminate between an isochronous and an irregular pulse pattern. Pigeons are vocal non-learners and this might be the reason for their inability, as it has been suggested that vocal learning and vocal non-learning species might differ in this respect (Patel et al., 2009; Schachner et al., 2009; see also ten Cate et al. (2016) for a review on this relationship for birds). The rhythm perception by zebra finches in the study by Benichov et al. seems to support this view, as they show that the (vocal learning) zebra finches extracted the regularity of the call pattern and used this to time their calling. However, when we combine these findings with several other recent studies on zebra finch perception of regularity the picture becomes more complex.

Nagel et al. (2010) trained female zebra finches to discriminate between two different songs and showed that the females maintained the discrimination over a range of tempo changes. The songs were still categorized correctly up to a 25% speed increase or decrease. These results might indicate rhythm generalization by zebra finches. However, Nagel et al.'s conclusion was that zebra finches maintained the discrimination by attending to the spectral envelope of the songs. Attending to the sequence of local spectral features, rather than any timing pattern may have enabled the discrimination. Whether zebra finches do attend to regularity in the timing of songs was examined by Lampen et al. (2014). They compared ZENK expression in response to playback of rhythmic zebra finch songs, i.e., where all songs in a string had identical inter-element intervals, with the expression in response to arrhythmic songs, i.e., a string of songs in which inter-element intervals vary. Arrhythmic songs resulted in stronger ZENK expression in the caudomedial nidopallium (NCM), the caudomedial mesopallium (CMM), and the nucleus taeniae (Tn). This increased activity in auditory areas of the zebra finch brain might be related to the finding by Benichov et al. (2016a) as, similar to their study, the repeated pattern in the regular song may have initiated predictive timing of the next song rendition, which would be lacking with the arrhythmic song.

## Regularity or interval detection?

The above mentioned studies may indicate that zebra finches perceive “regularity” as such. However, as also noticed by Benichov et al. (2016b) this need not be the case. The various findings may arise because repeated events with a fixed interval may give rise to a prediction for a next interval of the same absolute duration. So, when the birds respond to the robotic finch they can do so by attending to, and learning about, the absolute interval between successive robot calls, or by detecting other local contiguities of events (Benichov et al., 2016b), without having formed some concept of “regularity.” The same accounts for the study by Lampen et al. (2014), in which the zebra finches could also have responded to identical consecutive intervals in a sound string.

If zebra finches can perceive regularity as such, one would expect that they are not only able to distinguish a regular from an irregular string, but also to transfer this distinction to strings with modified tempos. This has been tested in experiments in which van der Aa et al. (2015) trained zebra finches to discriminate between a set of regular, isochronous pulse strings and a set of irregular pulse strings. The irregularity in these strings was created by varying the duration of inter-pulse-intervals within a string. As expected from the studies discussed above, zebra finches of both sexes could learn to discriminate these strings. But this discrimination broke down when the zebra finches were tested with probe strings with novel tempo transformations. The birds seemed to distinguish and discriminate the different strings based on their specific inter-pulse-interval durations, suggesting that the zebra finches focused on local features, without attending to or learning about the global pattern of regularity-irregularity of the strings (van der Aa et al., 2015).

Another recent experiment on zebra finches used isochronous pulse strings with pulses of two types. These were present in a ratio of 1:3, with the rare type raised in both frequency and amplitude compared to the other (ten Cate et al., 2016). This variation was used to create a string with a fixed number of low tones between two high ones, creating a regular beat pattern, and a string with different numbers of low tones in each interval, making the beat pattern irregular. Again, the zebra finches discriminated these strings. They were next tested with strings in which the location of the beat within the strings or the duration of pulses and inter-pulse-intervals was changed. This revealed that here also, the zebra finches seemed to use different local features, like the inter-beat- or inter-pulse-intervals to distinguish the strings. However, some birds seemed to combine a sensitivity to such local features with one for the more global regularity (ten Cate et al., 2016). Nevertheless, the behavioral experiments so far do not indicate that zebra finches perceive the global pattern of regularity as such. We should therefore be cautious with concluding that the ability of zebra finches (and other non-human species) to distinguish regular from irregular sound patterns is similar to the ability of humans to detect rhythm. However, there may be a continuum among species ranging from those not being able to discriminate regular from irregular sounds up those able of beat detection in more complex rhythms (ten Cate et al., 2016).

## Conclusion and outlook

The various studies discussed above show that zebra finches are very good at detecting fixed interval durations and can use preceding intervals to predict the next one in a string of sounds. This matches their ability to call and to produce songs with a fixed rhythmic periodicity. Furthermore, specific nuclei of the zebra finch forebrain song system (HVC, RA, CMM, and NCM) seem involved in producing and detecting rhythmicity. However, the results also call for further behavioral and neural studies on the links between perception and production of rhythmic patterns in the zebra finch. Are receivers sensitive to the rhythms underlying songs? And which brain areas are involved in the discrimination of the rhythmic patterns in the experiments of van der Aa et al. (2015) and ten Cate et al. (2016)? Are these perceptual abilities and also the production of the song rhythms observed by Norton and Scharff (2016) affected by interfering with HVC and RA? The zebra finch has proven to be an excellent model species, and is very suitable for addressing these questions. However, we also need comparative studies on other species to understand the types of temporal patterns that birds can detect, how they do so and how this is related to vocal learning (see also Benichov et al., 2016b). Ultimately, this may also shed light on the building blocks from which our human ability for rhythm perception and beat entrainment may have evolved.

## Funding

This work was supported by NWO-GW, grant no. 360.70.452.

### Conflict of interest statement

The authors declare that the research was conducted in the absence of any commercial or financial relationships that could be construed as a potential conflict of interest.
